# Impact on fetal growth of prenatal exposure to pesticides due to agricultural activities: a prospective cohort study in Brittany, France

**DOI:** 10.1186/1476-069X-9-71

**Published:** 2010-11-15

**Authors:** Claire Petit, Cécile Chevrier, Gaël Durand, Christine Monfort, Florence Rouget, Ronan Garlantezec, Sylvaine Cordier

**Affiliations:** 1 Inserm U625, GERHM, IFR140, Campus de Beaulieu, Rennes, F-35042 France; 2 IDHESA, Technopôle de Brest-Iroise, BP 52, 120 avenue A. de Rochon, 29280 Plouzané, France; 3 "Bien naître en Ille-et-Vilaine" Perinatal network, Aile de direction - Hôtel-Dieu CHU, 2 rue de l'Hôtel-Dieu, CS 26419, 35064 Rennes CEDEX, France; 4 Public Health department, CHU de Brest-Hôpital Morvan, Avenue Foch, 29200 Brest, France

## Abstract

**Background:**

Pesticide use is widespread in agriculture. Several studies have shown that pesticides used in agricultural fields can contaminate the domestic environment and thus be an important source of pesticide exposure of populations residing nearby. Epidemiological studies that have examined the health effects of *in utero *pesticide exposure from residence near agricultural activities suggest adverse effects, but the results are inconsistent. Our purpose was to investigate the effect on intrauterine growth of such exposure due to agricultural activities in the residential municipality.

**Methods:**

A prospective birth cohort recruited 3421 pregnant women in a French agricultural region (Brittany, 2002-2006) through gynecologists, ultrasonographers, and maternity hospitals during routine prenatal care visits before 19 weeks of gestation. The national agricultural census in 2000 provided the percentages of the municipality area devoted to cultivation of corn, wheat, colza, peas, potatoes, and fresh vegetables.

**Results:**

Birth weight and the risk of fetal growth restriction were not associated with agricultural activities in the municipality of residence in early pregnancy. Children whose mother lived in a municipality where peas were grown had a smaller head circumference at birth than those in municipalities not growing peas (-0.2 cm, p = 0.0002). Head circumference also tended to be lower when wheat was grown, but not to a statistically significant degree (p-trend = 0.10). Risk of an infant with a small head circumference was higher for mothers living in a municipality where peas (OR = 2.2; 95% CI = 1.2-3.6) or potatoes (OR = 1.5; 95% CI = 0.9-2.4) were grown.

**Conclusions:**

Agricultural activities in the municipality of residence may have negative effects on cranial growth. Cultivation of pea crops and, to a lesser degree, potato and wheat crops, may negatively affect head circumference. Insecticides, including organophosphate insecticides, were applied to most of the area devoted to pea and potato crops; this was less true for corn and wheat crops. These results must be interpreted in light of the study's limitations, in particular, the scale at which we could assess pesticide exposure.

## Background

Pesticides are used principally and massively in agriculture. After application, they enter the various environmental compartments: ground and surface water, soil, plants, and the atmosphere [1]. Several studies [2-4] have shown that pesticides used in agricultural fields and volatilized into the atmosphere contaminate the domestic environment and may therefore be an important source of pesticide exposure of local populations. Ward et al. [2] found that increasing acreage of corn and soybean fields near residences was associated with a greater probability of detecting one or more agricultural herbicides in house dust. Higher levels of pesticides in house dust [3] and higher levels of pesticide metabolites in children's urine [4] have been found in homes of agricultural workers closer to fields. In addition, some biological monitoring studies conducted in agricultural areas [5,6] have indicated fetal exposure to chemical pesticides, reporting residues of current-use and legacy pesticides in biological matrices such as maternal urine and amniotic fluid during pregnancy. Because their bodies are developing, infants exposed in utero and during early neonatal life are particularly vulnerable. Various epidemiological studies have examined the association between pesticide exposure of pregnant women living in agricultural areas and fetal growth [7-10]. Some of these suggest that this exposure has adverse effects on the intrauterine growth of their fetuses. The results are nonetheless inconsistent, and the studies are difficult to compare because of their different methods of exposure assessment: two studies used ecological data about agricultural pesticide use [7,8], one evaluated maternal history of pesticide exposure (combination of residential proximity to crops, household and spousal occupational exposure) [9], and another used urinary biomarkers of one specific chemical class of pesticides: organophosphate insecticides [10].

In 2004, France led Europe in pesticide sales with around 76,000 tons of active ingredients sold; and 90% of this was used for agricultural activities [11]. The Brittany region of France offers an excellent setting for studies of environmental exposure to pesticides, for 70% of the area is devoted to agricultural activities, in particular, grains for pig and poultry farming, vegetables, and potatoes. The purpose of this study was to determine whether prenatal environmental exposure to pesticides due to agricultural activities in the municipality of residence was associated with poor fetal growth in the PELAGIE cohort of pregnant women in Brittany.

## Methods

### Population and study design

PELAGIE is a prospective birth cohort designed to study the role of environmental pollutants on intrauterine and child development in three Breton districts (Ille-et-Vilaine, Côtes d'Armor, and Finistère). Gynecologists and ultrasonographers practicing in these three districts as well as midwives and obstetricians practicing in maternity hospitals in Côtes d'Armor were invited to participate. About 20% of the gynecologists and ultrasonographers and 3 maternity hospitals (of 5) in Côtes d'Armor volunteered to participate. From April 2002 through February 2006, they recruited pregnant women during routine prenatal care visits before 19 weeks of gestation. Each of them informed women about objectives and modalities of the PELAGIE study, asked her for consent, and gave her a questionnaire. Women completed this questionnaire at home and returned it to us, providing information about their family and individual social and demographic characteristics and lifestyle. We estimated the participation rate of women at 80%, from the number of returned questionnaires divided by the number of questionnaires provided to one physician's office (which included roughly 600 pregnant women). We then followed the women through delivery and obtained information about the pregnancy, delivery, and newborn's health from midwives, pediatricians, and hospital records. All participants provided informed consent, and the appropriate ethics committees approved the study.

During the enrollment period, 3592 women agreed to participate in this study, representing 3% of total births in the three Breton districts. We excluded 171 women who returned the questionnaire after 19 weeks of pregnancy. Information on pregnancy outcome was available for 3399 women; 22 were lost to follow-up. Moreover, for the present analyses, we excluded women with twin births (n = 39), fetal deaths (n = 39), those who lived outside the three districts (n = 111) or who had no identified municipality of residence (n = 11). Because we were studying environmental exposure to pesticides, we also excluded women who worked as farmers (n = 42). Thus the final sample analyzed 3159 women, living in 536 municipalities.

### Data on agricultural activities

Data on agricultural activities of the three study districts were collected from the national general census (RGA), as analyzed by the statistics department of the Ministry of Agriculture (AGRESTE). These data were provided by farmers, scaled at the level of municipalities, and updated in 2000. The census provided the percentage of area of the municipality used for agricultural activities according to crop, including corn, wheat, colza, peas, potatoes, and fresh vegetables (plus strawberries and melons). These data were matched with the mother's municipalities of residence in early pregnancy. The mean area of the municipalities in this study was 22 km^2 ^(range = 0.5 - 118 km^2^), and their mean population density 166 inhabitants per km^2 ^(range = 10 - 4217 inhabitants/km^2^).

### Fetal growth indicators

Fetal growth indicators, including weight and length at birth, and a cranial growth indicator such as head circumference at birth, were obtained from hospital records. Gestational age was assessed by the midwives or the obstetrician at delivery and took into account both the date of the last menstrual period and the results of the ultrasound examination during the first trimester of pregnancy (between 10 and 14 weeks, standard practice in France). Discrepancies were resolved by clinical judgment. Liveborn singleton children of the PELAGIE study without major malformations were classified with fetal growth restriction (FGR) if their birth weight was below the 5^th ^percentile of the distribution of expected birth weight, modeled according to gestational age (third-degree polynomial), sex, parity (third-degree polynomial), maternal weight (third-degree polynomial), height and age (second-degree polynomial) [12]. Newborns were classified with small head circumference (SHC) if their head circumference was below the 5^th ^percentile of the head circumference distribution at birth for a given gestational age and sex, according to the most recent French reference curves [13].

### Statistical analysis

We defined the level of urbanization of municipalities by a cutoff point of 20,000 inhabitants in the municipality in order to isolate the women living in the largest cities (n = 7) in Brittany. Those with fewer are referred to as "rural" and those with more as "urban", for simplicity's sake.

Tertiles were used to categorize as low, medium, and high the percentage of area used agriculturally (0-63%, 63-74%, > 74%) and the percentage of area used for corn crops (0-13%, 13-20%, >20%) and wheat crops (0-9%, 9-15%, >15%) in the municipalities where mothers lived in early pregnancy. Because of the small percentage of area used for colza, peas, potatoes, and other vegetables, we defined a dichotomous variable based on whether the crop was grown in the municipality at all.

Information on agricultural activities in municipalities is considered confidential and consequently was not provided when only one farmer in the municipality grew a given crop. The existence of confidential data was reported, however, meant that this crop was grown in the municipality but over an unknown area. Thus, for corn and wheat crops, the few municipalities (respectively n = 16 and n = 12) with confidential data were assigned to the low tertile, on the assumption that the percentage of area dedicated to these crops by only one farmer in a municipality was small. For the other crops (colza, peas, potatoes and fresh vegetables), municipalities with confidential data were assigned to the category "present" (respectively, n = 142, 142, 139, and 156 municipalities, home to n = 488, 712, 719 and 747 pregnant women).

Anova and logistic regression models were performed to study the risk factors for fetal growth outcomes. Multivariate linear and logistic regression models were used to test for associations between the level of urbanization and fetal growth outcomes and also to test for associations between agricultural activities in the municipality of residence for each type of crop and fetal growth outcomes. The last analyses were performed only in rural municipalities to avoid potential confounding bias specific to urban life. In light of the results, similar statistical analyses were conducted with municipalities grouped according to their agricultural activities. Covariate adjustments used in multivariate analyses were selected from a backward selection with a p-value less than 20%. The following covariates were considered: year of inclusion, district of residence at inclusion, maternal age (continuous), maternal educational level (primary education, some secondary education, and completed high school or higher), maternal alcohol consumption (never/occasionally, ≤ 1 drink per day, > 1 drink per day) and tobacco status at inclusion (non smoker, former smoker: smoked at conception but quit before inclusion, smoker), marital status, season in early pregnancy, high blood pressure during pregnancy, and gestational or preexisting diabetes. For weight and head circumference at birth we added as adjustment factors: pre-pregnancy body mass index (BMI) (continuous), maternal height (continuous), parity, child's sex, gestational age, and gestational age squared. For SHC we added: pre-pregnancy BMI (<18.5 kg/m^2^, 18.5-25 kg/m^2^, 25-30 kg/m^2^, ≥30 kg/m^2^), maternal height (<160 cm, 160-168 cm, ≥168 cm), and parity. Cesarean delivery was also added as an adjustment factor for head circumference outcomes. Based on previous results in the PELAGIE cohort [14], we conducted additional analyses adjusting for both shellfish and fish intake (not shown). Means or odds ratios (ORs) and their 95% confidence intervals (CI) were estimated.

Statistical tests for linear trend were conducted to assess dose-response relations as follows: after verifying the linearity hypothesis, we reported the p-value of the categorical variables (coded as 1, 2, and 3) declared in continuous in the multivariate linear and logistic regression models. We used SAS software, version 9.1 (SAS institute, Inc., Cary, NC) for all analyses.

## Results

Table 1 describes the sociodemographic characteristics of the population. Most of the women were recruited in the Ille-et-Vilaine district (66%) in 2003 and 2004 (73%). In all, 79% of the women lived in rural municipalities. Mean gestational age at inclusion was 11.7 weeks (range = 3-19 weeks), mean maternal age 30 years (range = 15.7-44.2 years) and mean pre-pregnancy BMI 22.5 kg/m^2 ^(range = 13.7-62.4 kg/m^2^). Educational level was high: 63% of women reported at least completing of high school; 14% smoked and 85% did not drink alcohol in early pregnancy; 45% were nulliparous (no previous live delivery).

**Table 1:** Description of sociodemographic characteristics of population (n = 3159)

Variable	N	%		Variable	N	%
**Child's sex**				**Maternal tobacco status in early pregnancy**		

Male	1613	51.1		Non smoker	2250	72.0

Female	1545	48.9		Former smoker	442	14.1

*Missing*	*1*			Smoker	433	13.9

**Level of urbanization**				*Missing*	*34*	

Urban	660	20.9		**Maternal alcohol consumption in early pregnancy**		

Rural	2499	79.1		Never/occasionally	2661	85.2

**Districts of inclusion**				≤ 1 drink/day	405	13.0

Ille-et-Vilaine	2076	65.7		> 1 drink/day	56	1.8

Côtes d'Armor - Finistère	1083	34.3		*Missing*	*37*	

**Year of inclusion**				**Parity**		

2002	388	12.3		0	1406	44.6

2003	1203	38.1		1	1171	37.2

2004	1110	35.1		≥ 2	572	18.2

2005 - beginning 2006	458	14.5		*Missing*	*10*	

**Gestational age at inclusion (weeks of amenorrhea) (mean (min-max))**	*11.7 (3.0-19.0)*			**Marital status**		

				Couple	3079	97.6

**Season in early pregnancy**				Single	75	2.4

Spring	768	24.3		*Missing*	*5*	

Summer	880	27.9		**Gestational or preexisting diabetes**		

Fall	737	23.3		No	2981	96.3

Winter	774	24.5		Yes	116	3.7

**Maternal age at inclusion (years)**				*Missing*	*62*	

< 25	345	10.9		**High blood pressure during pregnancy**		

25-29	1259	39.9		No	2931	94.4

30-34	1113	35.2		Yes	174	5.6

≥ 35	442	14.0		*Missing*	*54*	

**BMI before pregnancy (kg/m^2^)**				**Delivery with cesarean section**		

< 18.5	235	7.5		No	2553	82.6

18.5-25	2339	74.6		Yes	536	17.4

25-30	402	12.8		*Missing*	*70*	

≥ 30	159	5.1				

*Missing*	*24*					

**Maternal educational level**						

Primary education	578	18.3				

Some secondary education	579	18.5				

High school completed or higher	1995	63.3				

Missing	*7*					

Tables 2 and 3 summarize the risk factors for the four fetal growth indicators that we studied. As expected, birth weight and head circumference were both higher for boys than girls. The higher the maternal pre-pregnancy BMI and the higher the parity, the greater the baby's weight and head circumference at birth. The younger the mother, the smaller the baby (birth weight). Women with the lowest educational level (primary school) had smaller babies than women with more education (high school level or more). Birth weight or head circumference were also smaller for babies whose mothers smoked in early pregnancy (compared with non-smokers) or had high blood pressure. Other correlations showed that women with the lowest education level (primary school) were younger and smoked more during pregnancy. Women with cesarean deliveries had babies with a bigger head circumference or lower birth weight than women with vaginal deliveries.

**Table 2:** Description of risk factors related to birth weight and FGR status (n = 3159)

	Birth weight (g)	FGR (n = 172)	
	**Mean (95% CI)**	**N**	**OR (95% CI)**

**All *(std)***	3392 *(492.4)*		

**Child's sex**			

Male	3452 (3428-3476)**	87	Ref

Female	3330 (3305-3355)	85	1.0 (0.7-1.4)

*Missing*		*0*	

**Level of urbanization**			

Urban	3393 (3355-3532)	28	Ref

Rural	3392 (3372-3411)	144	1.4 (0.9-2.1)

**Districts of inclusion**			

Ille-et-Vilaine	3386 (3365-3408)	111	Ref

Côtes d'Armor - Finistère	3403 (3374-3433)	61	1.1 (0.8-1.5)

**Year of inclusion**			

2002	3381 (3331-3431)	23	1.1 (0.7-1.8)

2003	3381 (3353-3410)	64	Ref

2004	3390 (3361-3420)	56	0.9 (0.6-1.4)

2005 - beginning 2006	3436 (3390-3482)	29	1.2 (0.8-1.9)

**Season in early pregnancy**			

Spring	3406 (3371-3442)	34	Ref

Summer	3389 (3356-3422)	48	1.2 (0.8-1.9)

Fall	3380 (3343-3416)	42	1.3 (0.8-2.1)

Winter	3393 (3358-3429)	48	1.4 (0.9-2.2)

**Maternal age at inclusion (years)**			

< 25	3326 (3273-3379)**	21	1.4 (0.8-2.3)

25-29	3389 (3361-3416)	56	Ref

30-34	3403 (3374-3433)	70	1.4 (1.0-2.1)

≥ 35	3425 (3379-3472)	25	1.3 (0.8-2.1)

**BMI before pregnancy (kg/m^2^)**			

< 18.5	3226 (3162-3290)**	12	0.9 (0.5-1.7)

18.5-25	3389 (3368-3409)	128	Ref

25-30	3468 (3419-3516)	19	0.9 (0.5-1.4)

≥ 30	3500 (3423-3578)	13	1.5 (0.8-2.8)

*Missing*		*0*	

**Maternal educational level**			

Primary education	3339 (3298-3380)**	45	1.7 (1.1-2.4)

Some secondary education	3405 (3364-3446)	27	0.9 (0.6-1.4)

High school completed or higher	3404 (3382-3426)	100	Ref

*Missing*		*0*	

**Maternal tobacco status in early pregnancy**			

Non smoker	3403 (3382-3424)**	114	Ref

Former smoker	3446 (3400-3493)	18	0.8 (0.5-1.3)

Smoker	3284 (3237-3331)	40	1.9 (1.3-2.8)

*Missing*		*0*	

**Maternal alcohol consumption in early pregnancy**			

Never/occasionally	3389 (3370-3408)	146	Ref

≤ 1 drink/day	3420 (3372-3469)	18	0.8 (0.5-1.3)

> 1 drink/day	3340 (3208-3471)	5	1.7(0.7-4.3)

*Missing*		*3*	

**Parity**			

0	3313 (3287-3339)**	79	Ref

1	3435 (3407-3464)	70	1.1 (0.8-1.5)

≥ 2	3503 (3463-3544)	23	0.7 (0.4-1.1)

*Missing*		*0*	

**Marital status**			

Couple	3393 (3375-3411)	163	Ref

Single	3332 (3219-3446)	9	2.6 (1.3-5.3)

*Missing*		*0*	

**Gestational or preexisting diabetes**			

No	3392 (3374-3410)	161	Ref

Yes	3409 (3317-3500)	7	1.1 (0.5-2.5)

*Missing*		*4*	

**High blood pressure during pregnancy**			

No	3411 (3393-3429)**	143	Ref

Yes	3102 (3029-3176)	25	3.3 (2.1-5.3)

*Missing*		*4*	

**Cesarean delivery**			

No	3406 (3387-3425)**	131	Ref

Yes	3325 (3282-3367)	40	1.5 (1.0-2.2)

*Missing*		*1*	

* p < 0.1; ** p < 0.05

**Table 3:** Description of risk factors related to head circumference at birth and SHC status (n = 3159)

	Head circumference (cm)	SHC (n = 102)	
	**Mean (95% CI)**	**N**	**OR (95% CI)**

**All *(std)***	34.6 *(1.5)*		

**Child's sex**			

Male	34.9 (34.8-35.0)**	55	Ref

Female	34.4 (34.2-34.4)	47	0.9 (0.6-1.3)

*Missing*		*0*	

**Level of urbanization**			

Urban	34.6 (34.6-34.7)	16	Ref

Rural	34.7 (34.6-34.8)	86	1.4 (0.8-2.4)

**Districts of inclusion**			

Ille-et-Vilaine	34.6 (34.6-34.7)	69	Ref

Côtes d'Armor - Finistère	34.6 (34.6-34.7)	33	0.9 (0.6-1.4)

**Year of inclusion**			

2002	34.7 (34.5-34.8)	16	1.4 (0.7-2.5)

2003	34.6 (34.5-34.7)	37	Ref

2004	34.6 (34.5-34.7)	36	1.0 (0.6-1.6)

2005 - beginning 2006	34.6 (34.5-34.8)	13	0.9 (0.5-1.7)

**Season in early pregnancy**			

Spring	34.7 (34.5-34.8)	23	Ref

Summer	34.6 (34.5-34.7)	30	1.1 (0.6-2.0)

Fall	34.6 (34.5-34.7)	22	1.0 (0.5-1.8)

Winter	34.7 (34.6-34.8)	27	1.2 (0.7-2.1)

**Maternal age at inclusion (years)**			

< 25	34.5 (34.3-34.6)*	13	1.3 (0.7-2.4)

25-29	34.7 (34.6-34.7)	38	Ref

30-34	34.6 (34.5-34.7)	46	1.4 (0.9-2.1)

≥ 35	34.7 (34.6-34.9)	5	0.4 (0.1-0.9)

**BMI before pregnancy (kg/m^2^)**			

< 18.5	34.2 (34.0-34.4)**	14	1.9 (1.1-3.5)

18.5-25	34.6 (34.5-34.7)	76	Ref

25-30	34.8 (34.7-35.0)	9	0.7 (0.3-1.4)

≥ 30	35.1 (34.9-35.3)	3	0.6 (0.2-1.8)

*Missing*		*0*	

**Maternal educational level**			

Primary education	34.5 (34.4-34.6)	15	1.0 (0.6-1.7)

Some secondary education	34.7 (34.5-34.8)	20	0.8 (0.4-1.3)

High school completed or higher	34.6 (34.6-34.7)	67	Ref

*Missing*		*0*	

**Maternal tobacco status in early pregnancy**			

Non smoker	34.7 (34.6-34.7)**	66	Ref

Former smoker	34.7 (34.6-34.9)	14	1.1 (0.6-1.9)

Smoker	34.4 (34.2-34.5)	22	1.7 (1.1-2.9)

*Missing*		*0*	

**Maternal alcohol consumption in early pregnancy**			

Never/occasionally	34.6 (34.6-34.7)	91	Ref

≤ 1 drink/day	34.7 (34.6-34.9)	7	0.5 (0.2-1.1)

> 1 drink/day	34.8 (34.4-35.2)	3	1.6 (0.5-5.3)

*Missing*		*1*	

**Parity**			

0	34.5 (34.4-34.6)**	55	Ref

1	34.7 (34.6-34.8)	39	0.8 (0.5-1.3)

≥ 2	34.8 (34.7-34.9)	8	0.3 (0.2-0.74)

*Missing*		*0*	

**Marital status**			

Couple	34.6 (34.6-34.7)	100	Ref

Single	34.8 (34.4-35.1)	2	- ^a^

*Missing*		*0*	

**Gestational or preexisting diabetes**			

No	34.6 (34.6-34.7)	97	Ref

Yes	34.6 (34.3-34.9)	3	0.8 (0.2-2.5)

*Missing*		*2*	

**High blood pressure during pregnancy**			

No	34.7 (34.6-34.7)**	95	Ref

Yes	34.1 (33.8-34.3)	6	1.1 (0.5-2.5)

*Missing*		*1*	

**Cesarean delivery**			

No	34.6 (34.5-34.7)**	88	Ref

Yes	34.8 (34.7-34.9)	11	0.6 (0.3-1.1)

*Missing*		*3*	

^a^: No OR because too few cases (n < 3).

* p < 0.1; ** p < 0.05

Our population sample included 172 (5.4%) infants with FGR and 102 (3.2%) with SHC. The risk of FGR was higher for women with any of the following characteristics: the lowest educational level, smoked in early pregnancy, lived alone, had high blood pressure during pregnancy, or had a cesarean delivery. The risk of SHC was higher for women who smoked at inclusion or had a pre-pregnancy BMI less than 18.5 kg/m^2^. It was lower when parity was high (≥ 2) or the mother was older than 35 years.

Corn (319,608 hectares (ha); 16% of the area of the 3 Breton districts) and wheat (233,897 ha; 12%) were the most widespread crops in the study region. Colza grew in 75% of municipalities, but in small areas (on average, 1.6% of the municipality area). Peas, potatoes, and fresh vegetables were grown in less than half of municipalities. On average, pea crops covered respectively 1% (max: 7%) of the area of these municipalities, potatoes 1% (max: 26%), and fresh vegetables 2% (max: 48%).

The associations between agricultural activities in municipalities where mothers lived in early pregnancy and fetal growth outcomes are reported in tables 4 and 5. Level of urbanization was not associated with either birth weight or risk of FGR. Nor were birth weight or FGR status associated with agricultural activities in the mother's municipality in rural area. Like birth weight, length at birth was not associated with agricultural activities in rural municipalities of residence (data not shown). Infants born to women living in rural areas had a smaller head circumference at birth (-0.1 cm, p = 0.04) and an increased risk of SHC (OR = 1.8; 95% CI = 0.9-3.1; p = 0.06), on the borderline of significance. A statistically significant decreased head circumference at birth was observed for infants whose mother lived in a municipality where pea crops were grown (-0.2 cm, p = 0.0002). Children born to women living in a municipality with a high percentage of wheat crops had a smaller head circumference at birth. Women living in a municipality with pea crops had a risk twice as high of those in municipalities without pea crops of having an infant with a SHC (OR = 2.2; 95% CI = 1.2-3.6; p = 0.004). An increased risk of SHC, on the borderline of significance, was associated with potato crops in the mother's municipality of residence. Additional adjustment for shellfish and fish intake did not modify the results (data not shown). In light of the results, municipalities were grouped into 5 categories: (1) urban municipalities, rural municipalities (2) without pea and potato crops, (3) without pea crops but with potato crops, (4) with pea crops but without potato crops, and (5) with pea and potato crops.

**Table 4:** Relation between agricultural activities and birth weight and FGR status (n = 3159)

			**Birth weight (g) **^**a**^		**FGR (n = 172) **^**b**^			
**Variable**	**N**	**Number of municipalities**	**Mean (95% CI)**	**P-trend**	**Control**	**Case**	**OR (95% CI)**	**P-trend**

**Level of urbanization**								

Urban	660	7	3399 (3343-3455)	0.28	616	28	Ref	0.15

Rural	2499	529	3381 (3332-3430)		2314	144	1.4 (0.9-2.1)	

***In rural municipalities***								

**Percentage of area used agriculturally**								

Low	1039	177	3381 (3325-3438)	0.36	956	65	Ref	0.87

Medium	817	174	3375 (3317-3433)		759	43	0.9 (0.6-1.4)	

High	643	178	3363 (3303-3423)		599	36	1.0 (0.6-1.5)	

**Percentage of area used for corn**								

Low	990	183	3382 (3325-3438)	0.35	913	59	Ref	0.61

Medium	816	172	3375 (3316-3433)		759	45	1.0 (0.7-1.6)	

High	693	174	3364 (3305-3423)		642	40	1.1 (0.7-1.7)	

**Percentage of area used for wheat**								

Low	769	186	3375 (3319-3432)	0.66	714	45	Ref	0.66

Medium	842	172	3382 (3325-3439)		777	50	1.2 (0.8-1.8)	

High	888	171	3368 (3311-3424)		823	49	1.1 (0.7-1.7)	

**Colza crop**								

No	387	95	3391 (3327-3454)	0.35	364	19	Ref	0.14

Yes	2112	434	3370 (3316-3424)		1950	125	1.5 (0.9-2.5)	

**Pea crop**								

No	962	273	3381 (3325-3437)	0.47	895	50	Ref	0.12

Yes	1537	256	3369 (3314-3425)		1419	94	1.3 (0.9-1.9)	

**Potato crop**								

No	1340	282	3374 (3318-3430)	0.95	1239	74	Ref	0.94

Yes	1159	247	3375 (3319-3431)		1075	70	1.0 (0.7-1.4)	

**Vegetable crop**								

No	904	211	3389 (3331-3447)	0.19	846	46	Ref	0.59

Yes	1595	318	3368 (3314-3423)		1468	98	1.1 (0.8-1.6)	

^a^: All models adjusted for maternal tobacco status at inclusion, parity, child's sex, high blood pressure, gestational and pre-pregnancy diabetes, gestational age, gestational age squared, pre-pregnancy BMI and height of mother; ^b^: All models adjusted for maternal tobacco status at inclusion, maternal education level, high blood pressure during pregnancy and marital status.

**Table 5:** Relation between agricultural activities and head circumference at birth and SHC status (n = 3159)

			**Head circumference (cm) **^**a**^		**SHC (n = 102) **^**b**^			
**Variable**	**N**	**Number of municipalities**	**Mean (95% CI)**	**P-trend**	**Control**	**Case**	**OR (95% CI)**	**P-trend**

**Level of urbanization**								

Urban	660	7	34.9 (34.7-35.1)	0.04	627	16	Ref	0.06

Rural	2499	529	34.8 (34.6-35.0)		2374	86	1.8 (0.9-3.1)	

***In rural municipalities***								

**Percentage of area used agriculturally**								

Low	1039	177	35.0(34.7-35.1)	0.36	986	39	Ref	0.35

Medium	817	174	34.9 (34.7-35.1)		772	31	1.1 (0.6-1.8)	

High	643	178	34.9 (34.7-35.0)		616	16	0.7 (0.3-1.2)	

**Percentage of area used for corn**								

Low	990	183	35.0 (34.7-35.1)	0.38	939	35	Ref	0.94

Medium	816	172	34.9 (34.7-35.1)		777	29	1.1 (0.6-1.8)	

High	693	174	34.9 (34.7-35.0)		658	22	1.0 (0.5-1.7)	

**Percentage of area used for wheat**								

Low	769	186	35.0 (34.8-35.1)	0.10	740	21	Ref	0.17

Medium	842	172	35.0 (34.7-35.1)		794	33	1.6 (0.8-2.7)	

High	888	171	34.9 (34.7-35.0)		840	32	1.5 (0.8-2.7)	

**Colza crop**								

No	387	95	35.0 (34.8-35.2)	0.14	372	8	Ref	0.13

Yes	2112	434	34.9 (34.7-35.0)		2002	78	1.8 (0.8-3.7)	

**Pea crop**								

No	962	273	35.1 (34.9-35.2)	0.0002	927	20	Ref	0.004

Yes	1537	256	34.9 (34.6-35.0)		1447	66	2.2 (1.2-3.6)	

**Potato crop**								

No	1340	282	35.0 (34.7-35.1)	0.38	1274	40	Ref	0.07

Yes	1159	247	34.9 (34.7-35.0)		1100	46	1.5 (0.9-2.4)	

**Vegetable crop**								

No	904	211	35.0 (34.8-35.1)	0.12	860	27	Ref	0.40

Yes	1595	318	34.9 (34.7-35.0)		1514	59	1.2 (0.7-1.9)	

^a^: Models adjusted for maternal tobacco status and alcohol consumption at inclusion, parity, child's sex, cesarean delivery, gestational age, gestational age squared, pre-pregnancy BMI and height of mother. In addition, for level of urbanization, model adjusted for high blood pressure and season and without maternal alcohol consumption. District of residence and season included as additional adjustment for pea crop; ^b^: Models adjusted for maternal age at inclusion, maternal tobacco status, parity, cesarean delivery, pre-pregnancy BMI and height of mother. Maternal alcohol consumption included as additional adjustment for the level of urbanization and year of inclusion for potato crop

We observed no statistically significant differences in risk for SHC between women living in urban municipalities (reference category) and women living in rural municipalities without pea and potato crops (OR = 0.7; 95% CI = 0.3-1.8) or without peas but with potatoes (OR = 1.3; 95% CI = 0.6-2.9). Women living in a rural municipality with pea crops, regardless of the presence of potato crops, had an increased risk of having an infant with a SHC (without potato crops: OR = 1.9; 95% CI = 1.0-3.7; with potato crops: OR = 2.6; 95% CI = 1.4-5.0). A similar grouping of municipalities was established for pea and wheat crops for testing their association with head circumference at birth. Similar mean head circumference at birth was observed for the babies of women living in urban municipalities (Reference category, mean = 34.9 cm; 95% CI = 34.7-35.1 cm) and rural municipalities without pea crops and with a small area of wheat crops (1^st ^tertile) (mean = 34.9 cm; 95% CI = 34.7-35.1 cm) or without pea crops but with a high area devoted to wheat crops (2^nd ^and 3^rd ^tertiles) (mean = 34.8 cm; 95% CI = 34.6-35.0 cm). Women residing in rural municipalities with pea crops, regardless of the wheat-growing area, had infants with a smaller head circumference at birth (small wheat area: mean = 34.6 cm; 95% CI = 34.4-34.8 cm; high wheat area: mean = 34.7 cm; 95% CI = 34.6-34.9 cm).

## Discussion

We investigated the impact of environmental exposure to agricultural pesticides during pregnancy on fetal growth in Brittany from 2002 to 2006. This study suggested that the babies of women living in rural municipalities had a smaller head circumference at birth as well as an increased risk of SHC. A decreased head circumference at birth and an increased risk of SHC were observed with the presence of pea crops in the rural municipality of residence. Similar borderline significant associations between head circumference outcomes and other agricultural activities were suggested: the cultivation of potatoes and wheat in the municipality where the mother resided in early pregnancy.

In 2003, according to a national perinatal survey [15], pregnant women in France were on average 29 years, 44% were nulliparous, 43% had at least completed high school, and 75% did not smoke in the third trimester of pregnancy. The women of the PELAGIE study, representing a small proportion of the population of pregnant women in Brittany were better educated and smoked less than the national population of pregnant women. Despite this enrollment bias, which suggests that the women included in our study were probably healthier, the study observed known risk factors for birth weight and head circumference outcomes.

The main limitation of our study is that the assessment of residential proximity to crops depends on the scale of our agricultural activity data -- the municipality. Pesticides applied to crops can travel through air, by processes such as spray drift [16] and also post-application volatilization, sometimes for substantial distances, on the order of 10 km or more [17]. In the 3 districts under study, the mean size of a municipality was 22 km^2 ^(range = 0.5 - 118 km^2^). Thus even women living quite far away of crops of residence municipality may have been exposed to pesticides applied to crops of their residence municipality.

Corn, wheat, colza and pea cultivation were highly intercorrelated, as were potato and vegetable production. That is, in a municipality where corn is grown, it is likely that wheat, colza, and pea crops are also grown. The national general census provided us with a variety of data about crop types. However, it is difficult to attribute to a specific crop the responsibility for the adverse associations suggested between fetal growth and pea, potato, and wheat production. Our agricultural activity data were based on the 2000 census, and the study began in 2002 and finished at the beginning of 2006. Nevertheless, the soil occupancy did not change substantially between 2000 and 2008 [18]. Another limitation is the possibility of misclassification of exposure for mothers who moved during pregnancy. In a questionnaire sent to the families when the child was two years old, we asked for a list of their residences during pregnancy and can thus estimate that overall 7% of women moved while pregnant from their municipality of residence. A final limitation involves the confidentiality of some agricultural data. We assigned municipalities with a confidential value for the cultivation of corn and wheat to the first tertile, assuming that the percentage of area devoted to these crops by only one farmer in a municipality was small. The associations between the percentages of area of corn or wheat crops and birth outcomes did not change more than 1% if we removed the women living in the municipalities with confidential data for corn and wheat crops from the analysis.

As mentioned above, four epidemiological studies have looked at the effects on fetal growth of prenatal pesticide exposure from agricultural activities, with inconsistent results. Concerning birth weight, the Colorado study [7] suggested an inverse association with nearby production of sugar beets and corn during pregnancy. In California, Eskenazi et al. [10], using urinary biomarkers of exposure to organophosphate pesticides, found no association with birth weight. A study [9] in Mexico showed an increased risk of intrauterine growth retardation with the mother's history of pesticide exposure (combination of residential proximity to agricultural communities, pesticide handling by household members, and a spouse working in agriculture). Schreinemachers [8] found no adverse relation between the wheat acreage and the risk of small-for-gestational-age births. We did not find a statistically significant association between birth weight and agricultural activities in the municipality of residence. In particular, our results about birth weight and local corn crops differ from those of Xiang et al. [7]. Like Schreinemachers [8], we found that the risk of FGR was not associated with wheat crops in the municipality of residence.

Eskenazi et al. [10] assessed the potential impact in an agricultural area of organophosphate insecticide exposure on head circumference at birth. They found that head circumference increased with the level of dialkyl phosphate metabolites in maternal urine in early pregnancy. In addition, two other studies in rural areas [19,20], studied the effect of organophosphate insecticide exposure on head circumference at birth. Whyatt et al. [20] observed no statistically significant decrease in head circumference at birth with organophosphate insecticide concentrations in personal air samples or cord plasma samples. Berkowitz et al. [19] reported a significant reduction in head circumference with the level of organophosphate insecticides in maternal urine, but only among women with low PON1 activity. In the present study, a decreased head circumference at birth and an increased risk of SHC were observed among babies of women living in rural municipalities in which a higher exposure to agricultural pesticides was expected, compared to urban ones. Our overall results suggest that this association may be explained by the presence of pea crops in the municipality of residence of women in early pregnancy, and, to a lesser degree, potato and wheat crops. This finding in turn suggests that specific pesticide mixtures used on these crops may play a role. According to a national survey on agricultural practices conducted in France in 2001 and 2006, insecticides are applied to most of the area used to grow potatoes, peas, and colza, but to only a small proportion of the area devoted to corn and wheat. Similarly, these crops also received more insecticide treatments than corn and wheat crops [21,22]. More especially, over the study period (2002-2006), organophosphate insecticides was authorized for use on peas (7 chemicals), potatoes (6 chemicals) and, to a lesser degree, fresh vegetable (3 chemicals), and grain (2 chemicals) crops. Although we did not know the real uses of these insecticides on crops in Brittany, our results may provide evidence consistent with the potential impact of organophosphate insecticides on head circumference suggested by the American cohorts [19,20].

## Conclusion

This study suggests that some adverse effects on cranial growth during fetal development are caused by prenatal exposure to agricultural activities in the municipality of residence, especially pea crops. This may be a cause for concern, as the cranial growth may be predictive of IQ and cognitive ability. These results must be interpreted in light of the study's limitations, in particular, the scale according to which pesticide exposure was assessed. It would be interesting to complete or confirm the results of this study by using the GIS method to take into account the proximity of agricultural activities, that is, to measure the distance between the women's homes and the crops.

## Abbreviations

FGR: Fetal Growth Restriction; SHC: Small Head Circumference

## Competing interests

The authors declare that they have no competing interests.

## Authors' contributions

CP performed statistical analysis, was involved in interpretation of the results, and drafted the manuscript. CC oversaw statistical analysis, was involved in interpretation of the results, and helped to draft and revise the manuscript. GD provided data on agricultural activities from the national agricultural census and helped in interpretation of the results. CM assisted in the planning of the study, data collection, and data management. FR was the pediatrician coordinator and was involved in study planning and medical data collection. RG was in charge of medical data collection, coding and quality control. SC conceived and designed the study, supervised the overall project and was involved in the interpretation of the results and revision of the paper. All authors read and approved the final manuscript.
